# Human Amniotic Epithelial Stem Cells and Osteoblast Cells Behavior on Collagen Membranes for Bone Guided Regeneration

**DOI:** 10.3390/ijms27156660

**Published:** 2026-07-26

**Authors:** Antonio Pérez-Pérez, Javier Gil, Isabela Bueno-Bianchi, Loreto Monsalve-Guil, Iván Ortiz-Garcia, Alvaro Jiménez-Guerra, Enrique Núñez-Márquez, Eugenio Velasco-Ortega, José Luis Rondón Romero, Victor Sánchez-Margalet, Jesús Moreno-Muñoz

**Affiliations:** 1 Departamento de Bioquímica Médica y Biología Molecular e Inmunología, Hospital Universitario Virgen Macarena, Facultad de Medicina, Universidad de Sevilla, Avenida Sánchez Pizjuán 4, 41009 Sevilla, Spain; aperez14@us.es (A.P.-P.); margalet@us.es (V.S.-M.); 2 Biomaterials i Interfícies Orals Bioinspirats (BOBI), Departament de Ciència i Enginyeria de Materials i Centre de Recerca en Ciència i Enginyeria Multiescala de Barcelona (CCEM), Escola d’Enginyeria de Barcelona Est (EEBE), Universitat Politècnica de Catalunya · BarcelonaTech (UPC), Av. Eduard Maristany, 16, 08019 Barcelona, Spain; 3 Department of Stomatology, Faculty of Dentistry, Universidad de Sevilla, 41004 Sevilla, Spain; isabela.buenobianchi@hotmail.com (I.B.-B.); lomonsalve@hotmail.es (L.M.-G.); ivanortizgarcia1000@hotmail.com (I.O.-G.); alopajanosas@hotmail.com (A.J.-G.); enrique_aracena@hotmail.com (E.N.-M.); jolurr001@hotmail.com (J.L.R.R.); je5us@hotmail.com (J.M.-M.)

**Keywords:** bone regeneration, collagen membranes, human amniotic epithelial cells, osteoblasts, tissue engineering, guided bone regeneration, osteogenesis

## Abstract

Guided bone regeneration (GBR) heavily relies on barrier membranes, with collagen being the clinical standard material. Human amniotic epithelial cells (hAECs) represent a promising, non-controversial stem cell source with substantial osteogenic and immunomodulatory potential. This study aimed to comparatively evaluate the structural characteristics of three commercial collagen membranes (Biocollagen^®^, Derma^®^, and VantyColl^®^) and their influence on the biological behavior, viability, and osteogenic differentiation of hAECs and hFOB 1.19 human fetal osteoblasts. The microarchitecture was assessed via scanning electron microscopy (SEM). Biological response was evaluated over 14 days, using MTT assays, calcium and phosphorus quantification, alkaline phosphatase (ALP) activity, and quantitative real-time PCR (qRT-PCR) for osteogenic markers (*Runx2*, *Osterix*, ALP, and *OPN*). SEM revealed a dense lamellar structure for Biocollagen^®^, a fibrillar and oriented architecture for Derma^®^, and a highly porous network for VantyColl^®^. Both cell types adhered to and proliferated on all membranes. Derma^®^ provided the best long-term proliferative support for both lineages. Conversely, VantyColl^®^ induced robust early osteoblastic differentiation, marked by exceptional upregulation of *Osterix* (24.93-fold) and *Runx2* (2.64-fold), though it exhibited diminished long-term hAEC viability. Ultimately, collagen membrane microarchitecture dictates cell fate; dense fibrillar networks (Derma^®^) favor sustained growth and late matrix maturation (*OPN*), whereas high-porosity scaffolds (VantyColl^®^) amplify early osteoinductive cascades.

## 1. Introduction

Guided bone regeneration (GBR) has become a cornerstone technique in implant dentistry for the reconstruction of alveolar bone deficiencies prior to or simultaneously with implant placement. Successful GBR procedures rely on the coordinated interaction between biological processes and biomaterial properties, ultimately enabling the restoration of adequate bone volume and architecture for long-term implant stability. The clinical outcomes of GBR are strongly influenced by the characteristics of both barrier membranes and grafting materials, which differ in their osteogenic, osteoinductive, and osteoconductive potential, as well as in their resorption kinetics, mechanical strength, and volumetric stability. By creating a protected environment that excludes competing soft tissue cells while supporting bone-forming cell activity, GBR promotes predictable regeneration in compromised sites and expands treatment possibilities for patients presenting with severe alveolar bone loss [1,2,3].

Bone regeneration is a complex biological process involving a cascade of cellular and molecular events, including inflammation, angiogenesis, recruitment of progenitor cells, extracellular matrix deposition, and bone remodeling. A critical factor in this process is the maintenance of a secluded space where osteogenic cells can proliferate and differentiate without interference from rapidly growing connective tissue. For this reason, the use of barrier membranes has become an essential component of GBR procedures. Since their introduction, collagen-based membranes have gained widespread acceptance due to their excellent biocompatibility, low immunogenicity, favorable tissue integration, and ability to undergo physiological degradation without requiring surgical removal [4,5].

From a biological perspective, collagen membranes must fulfill several functional requirements. They should effectively prevent the migration of epithelial cells and fibroblasts into the defect area while simultaneously allowing the diffusion of oxygen, nutrients, and signaling molecules required for tissue regeneration. In addition, they must provide sufficient mechanical stability to preserve the regenerative space throughout the early phases of healing. Ideally, these membranes exhibit a bilayered structure consisting of a dense surface that acts as a cellular barrier and a porous surface that facilitates cell attachment, vascular infiltration, and new bone formation [6,7]. The degradation profile of collagen membranes is equally important, as premature degradation may compromise regenerative outcomes, whereas excessive persistence could interfere with physiological tissue remodeling. Therefore, a degradation period ranging from three to six months is generally considered desirable to match the timeline of new bone formation [8].

Beyond their barrier function, increasing evidence suggests that the physicochemical characteristics of collagen membranes play a direct role in regulating cellular behavior. Surface topography, roughness, porosity, stiffness, wettability, and fiber organization can significantly influence cell adhesion, proliferation, migration, and differentiation. These properties affect the interactions between biomaterials and the surrounding biological environment by modulating protein adsorption, cell–matrix communication, and mechanotransduction pathways. Consequently, membrane architecture is now recognized as an active determinant of tissue regeneration rather than merely a passive physical barrier [8,9]. Understanding how specific membrane features influence the biological response of osteogenic cells is therefore essential for the development of next-generation GBR materials with enhanced regenerative potential.

Despite the widespread clinical use of collagen membranes in GBR, considerable heterogeneity exists among commercially available products regarding collagen source, processing methods, cross-linking approaches, fiber organization, thickness, porosity, and surface architecture. These structural differences may substantially influence membrane degradation kinetics, mechanical performance, protein adsorption, and cellular responses. Although several studies have characterized the physicochemical properties of collagen membranes, the relationship between membrane microstructure and the biological behavior of osteogenic cells remains incompletely understood. In particular, comparative investigations involving multiple commercially available membranes are limited, and the extent to which their topographical characteristics modulate cell viability and osteogenic differentiation has yet to be fully elucidated. Therefore, a better understanding of how membrane architecture influences cellular responses is essential for optimizing membrane selection and improving regenerative outcomes in GBR procedures.

In parallel with advances in biomaterial design, tissue engineering strategies have increasingly focused on combining scaffolds with stem cell-based therapies to improve bone regeneration. Among the various stem cell populations investigated, human amniotic epithelial cells (hAECs), isolated from the innermost layer of the placenta, have emerged as a particularly attractive cell source. These cells can be obtained through non-invasive procedures, avoiding ethical concerns associated with embryonic stem cells while providing an abundant and readily available cell population [10,11]. Furthermore, hAECs exhibit low immunogenicity, potent immunomodulatory activity, anti-inflammatory effects, and the ability to secrete a wide range of bioactive molecules involved in tissue repair and angiogenesis.

Importantly, hAECs possess multilineage differentiation potential and have demonstrated the capacity to differentiate toward osteogenic, chondrogenic, adipogenic, and neurogenic lineages under appropriate culture conditions [12,13]. Previous studies have highlighted their potential application in bone tissue engineering, where they may contribute not only through direct differentiation into osteoblast-like cells but also through paracrine mechanisms that stimulate endogenous regeneration. Nevertheless, the interaction between hAECs and commercially available collagen membranes remains incompletely understood. In particular, little information is available regarding how membrane microstructure and surface topography influence the biological performance of hAECs compared with mature osteoblasts.

Therefore, the present study aimed to comparatively evaluate the structural characteristics of collagen membranes exhibiting different microarchitectural and topographical features and to investigate their influence on the biological behavior of human amniotic epithelial cells (hAECs) and human osteoblasts. Particular attention was given to the relationship between membrane surface characteristics and cellular responses, including viability and osteogenic differentiation. By correlating structural membrane properties with osteogenic performance, this study seeks to provide insights into the role of membrane architecture in guided bone regeneration and to identify biomaterial features that may contribute to improved regenerative outcomes.

## 2. Results

### 2.1. Characterization of the Collagen Membranes

SEM (scanning electron microscopy) analysis revealed significant morphological differences between the tested materials at the qualitative level; systematic morphometric quantification (pore size distribution, fiber diameter, porosity percentage) was outside the scope of the present study and is discussed as a limitation below.

#### 2.1.1. Biocollagen^®^, Bioteck (Arcugnano, Italy)

**Figure 1:**
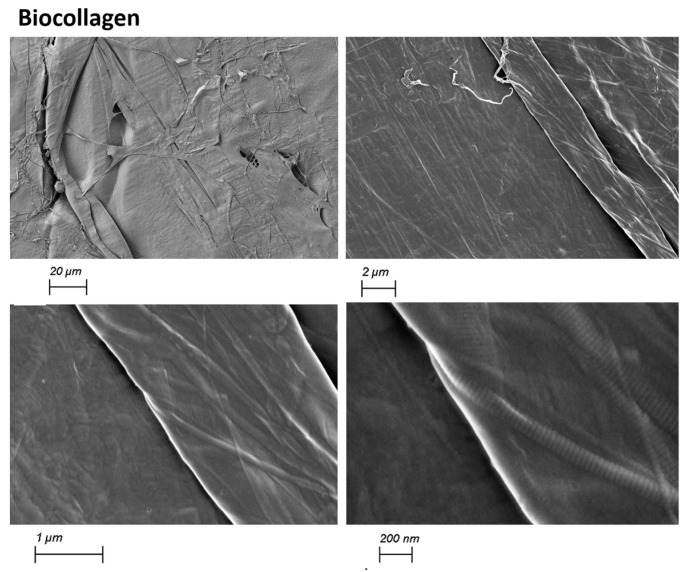
This membrane exhibited a lamellar morphology with a remarkably dense and compact structure. Higher magnification micrographs revealed a relatively smooth surface and low visible porosity.

This membrane exhibited a lamellar morphology with a remarkably dense and compact structure, even at magnifications of 350× and 3.00 K×. Higher magnification micrographs (12.00 K× and 30.00 K×) revealed a relatively smooth surface and low visible porosity, suggesting an organization of collagen fibers in the form of tightly bound sheets (Figure 1).

#### 2.1.2. Derma^®^, Osteobiol (Tecnos, Giaveno, Italy)

**Figure 2:**
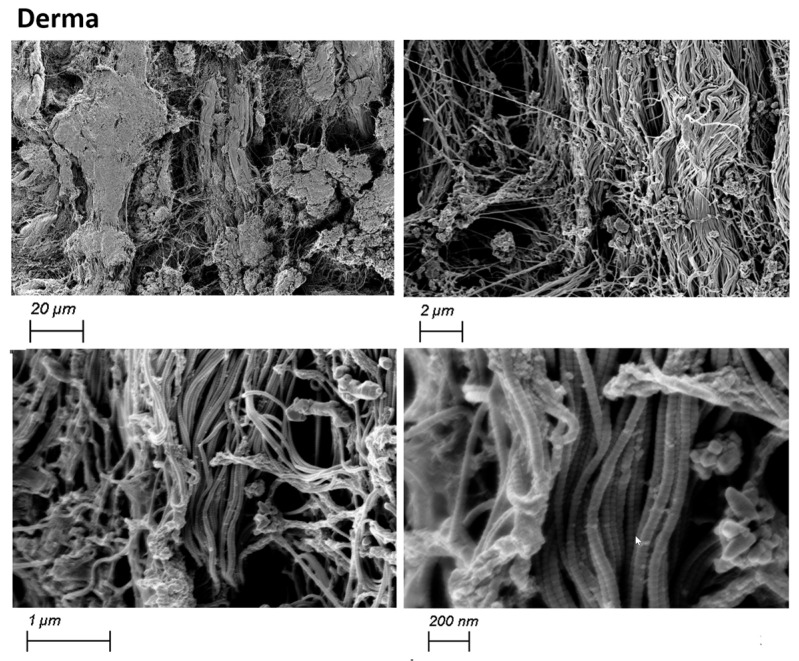
This membrane exhibited a distinctly fibrillar morphology. At increasing magnification, it is composed of a bundle of oriented and tightly packed collagen fibers.

This membrane exhibited a distinctly fibrillar and three-dimensional architecture. At increasing magnification, it was observed that the material is composed of a bundle of oriented and tightly packed collagen fibers. This organization results in high structural density, but with a rough surface morphology due to the arrangement of the microfibers. (Figure 2).

#### 2.1.3. VantyColl^®^, Techbiomat (Barcelona, Spain)

**Figure 3:**
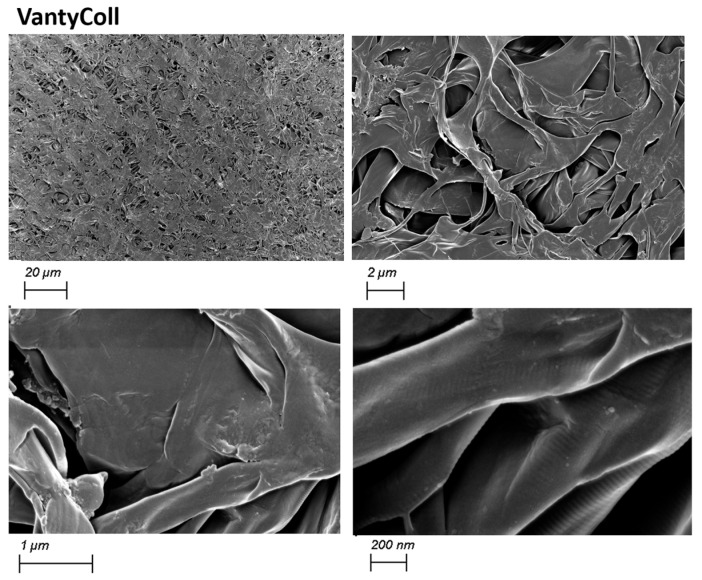
This membrane exhibited a porous morphology, characterized by a fibrous network that creates large interconnected spaces. At low magnifications, it is defined as having high porosity. A higher resolution confirms the presence of collagen fibers.

This membrane exhibited a porous and open morphology, characterized by a fibrous network that creates large interconnected spaces. At low magnifications, a network of disordered fibers is observed, defining the high porosity. Higher-resolution micrographs confirm the presence of collagen fibers forming this scaffold with a highly rough structure at the submicron level (Figure 3).

### 2.2. Biological Characterization of Collagen Membranes

#### 2.2.1. Cell Viability

##### Human Amniotic Epithelial Stem Cells (hAECs)

**Figure 4:**
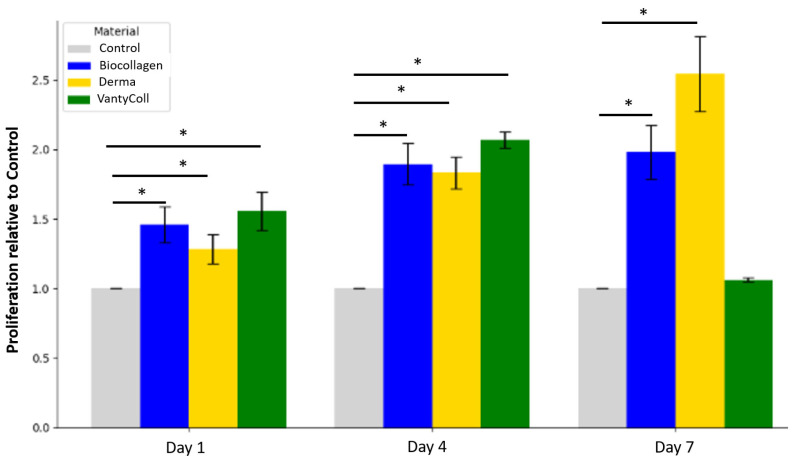
Analysis of the proliferation of human amniotic epithelial cells (hAECs; n = 4 independent biological experiments per group) cultured on collagen membranes (Biocollagen^®^, Derma^®^, and VantyColl^®^) versus control at 1, 4, and 7 days. Cell viability was determined by MTT assay, and data are presented as relative proliferation normalized to the control group (value 1.0). Bars represent the mean ± standard deviation. The asterisk (*) denotes statistically significant differences compared to the control (*p* < 0.05).

The proliferative response of hAECs on the different membranes was monitored for 7 days (Figure 4). Unlike osteoblast cultures, hAECs showed distinct growth dynamics depending on the exposure time and membrane type. In the initial stage (day 1), a favorable response was observed in all experimental groups. All three membranes (Biocollagen^®^, Derma^®^, and VantyColl^®^) promoted significantly greater metabolic activity than the control (*p* < 0.05).

At this point, VantyColl^®^ and Biocollagen^®^ showed the highest mean values, with relative proliferation close to 1.5 times the control value. On day 4, the growth trend remained positive for all materials. Significant increases compared to the control were recorded in all three groups. Specifically, the VantyColl^®^ group reached its peak performance on this day, exceeding a relative value of 2.0, followed closely by Biocollagen^®^ and Derma^®^, demonstrating that up to this point the membranes adequately supported cell expansion.

However, by day 7, a notable change in the materials’ behavior was evident. While Derma^®^ continued to promote exponential cell growth, reaching the highest value of the entire study (approximately 2.5), and Biocollagen^®^ maintained significant proliferation (~2.0), the VantyColl^®^ group experienced a decline. Proliferation in VantyColl^®^ fell to baseline levels similar to the control (~1.05) and, unlike previous days, showed no statistically significant differences compared to the control.

##### hFOB 1.19 Osteoblasts

**Figure 5:**
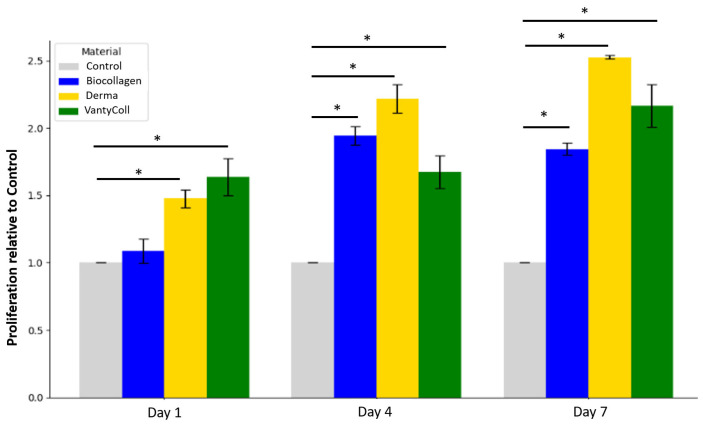
Evaluation of hFOB 1.19 osteoblast proliferation (n = 4 independent biological experiments per group) cultured on different collagen membranes (Biocollagen^®^, Derma^®^, and VantyColl^®^) compared to the control (no membrane) at 1, 4, and 7 days of culture. Cell proliferation was quantified using the MTT assay, and results are expressed as proliferation relative to the control (normalized to 1.0). Error bars represent standard deviation. The asterisk (*) indicates statistically significant differences compared to the control group (*p* < 0.05).

The viability and proliferation of hFOB 1.19 human fetal osteoblasts (ATCC^®^ CRL-11372, Diatek, Kalyani, India) on the different collagen membranes were evaluated using the MTT colorimetric assay at 1, 4, and 7 days (Figure 5). As shown in the figure, all the tested collagen membranes allowed cell growth, showing values equal to or higher than the control at all time points.

In the early adhesion and proliferation stage (Day 1), the Derma^®^ and VantyColl^®^ membranes showed a significant increase in metabolic activity compared to the control (*p* < 0.05), reaching approximate relative values of 1.5 and 1.6, respectively. In contrast, the Biocollagen^®^ group did not show significant differences compared to the control in these first 24 h. From Day 4 onward, a marked proliferative effect was observed in all the tested biomaterials. Biocollagen^®^, Derma^®^, and VantyColl^®^ all exhibited significantly higher proliferation rates than the control (Figure 5).

It is worth noting that the Derma^®^ group exhibited the highest average proliferation at this time point, exceeding a relative value of 2.0. This trend continued and strengthened by Day 7. At this final point, all experimental groups showed statistically significant differences compared to the control. The Derma^®^ group stood out notably, reaching the highest proliferation peak of the entire experiment (approximately 2.5 times higher than the control). The VantyColl^®^ group also showed high performance (>2.0), followed by Biocollagen^®^ (~1.8) (Figure 5).

#### 2.2.2. Calcium and Phosphorus Levels and Alkaline Phosphatase Activity

**Figure 6:**
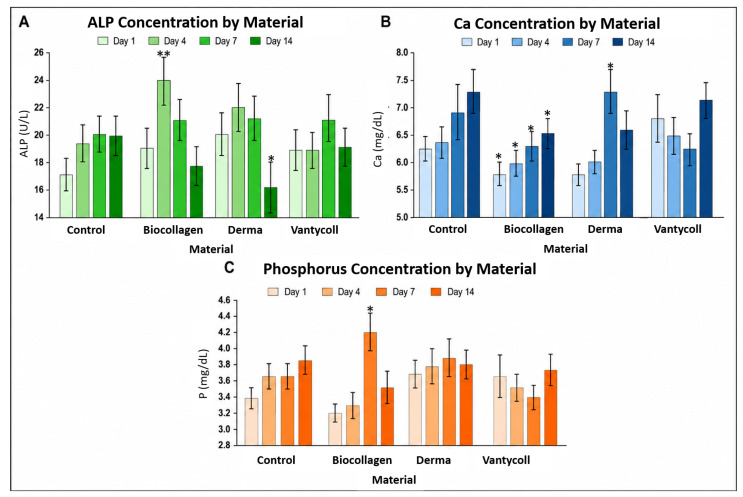
Quantification of calcium (Ca), phosphorus (P), and alkaline phosphatase (ALP) levels in conditioned medium of human amniotic epithelial cells (hAECs; n = 4 independent biological experiments per group). Culture supernatants were collected at 1, 4, 7, and 14 days of interaction with collagen membranes (Biocollagen^®^, Derma^®^, VantyColl^®^) and as a control. Bars represent mean concentration ± standard deviation. Statistical comparisons were performed by one-way ANOVA with Tukey’s post hoc test at each time point (and two-way ANOVA for the overall membrane × time effect, Section 4.8); asterisks denote significant differences vs. control (* *p* < 0.05, ** *p* < 0.01). Note the differences in the Y-axis scales between the two cell types, due to the substantially different concentration ranges observed between hAECs and osteoblastic cells; within each panel, the scale is consistent across all groups compared. An asterisk (*) indicates statistically significant differences (*p* < 0.05) compared to those without an asterisk or those with a double asterisk (**). A double asterisk indicates statistically significant differences compared to those without an asterisk or those with only a single asterisk (*).

As shown in Figure 6, the dynamics of calcium (Ca), phosphorus (P), and enzyme activity (alkaline phosphatase, PALK) levels varied considerably among cell lines and collagen membranes.

In hAEC cultures, material-specific fluctuations in Ca and P were observed. Regarding calcium, the Derma^®^ group showed a marked release peak on day 7, significantly higher than the other groups, followed by a decrease on day 14. As for phosphorus, the Biocollagen^®^ group showed a drastic and transient increase on day 7, suggesting active release of phosphate ions during this intermediate stage. ALP activity in hAECs showed a general tendency to reach its maximum in the early stages (day 4) for the Biocollagen^®^ and Derma^®^ membranes, subsequently decreasing, while VantyColl^®^ maintained high ALP levels until day 7 (Figure 6).

**Figure 7:**
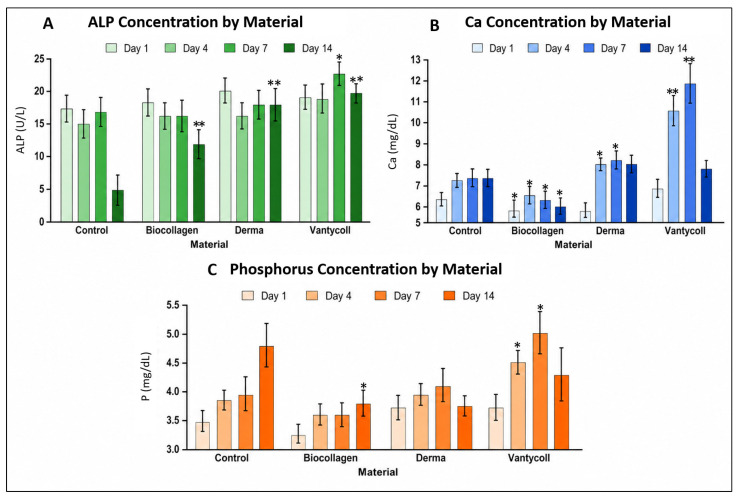
(**A**). Quantification of alkaline phosphatase (ALP) (**B**). calcium (Ca), (**C**). phosphorus (P) levels in conditioned medium of hFOB 1.19 osteoblasts (n = 4 independent biological experiments per group). Culture supernatants were collected at 1, 4, 7, and 14 days of interaction with collagen membranes (Biocollagen^®^, Derma^®^, VantyColl^®^) and as a control. Bars represent mean concentration ± standard deviation. Note the differences in the *Y*-axis scales between the two cell types. * *p* < 0.05, ** *p* < 0.01.

In osteoblastic cells, the behavior was different (Figure 7). The VantyColl^®^ group stood out from the others by inducing the highest concentrations of Ca and P in the medium, reaching their peak on day 7. This simultaneous increase in Ca and P suggests an active interaction of the material with osteoblastic mineral metabolism. Conversely, the Biocollagen^®^ group showed low baseline levels of Ca and P throughout the experiment, even lower than the control, which could indicate capture or precipitation of these ions onto the membrane (mineralization). Finally, ALP activity in osteoblasts was sustained over time, with VantyColl^®^ again standing out with the highest values on day 7, coinciding with its peak Ca and P levels, suggesting a strong stimulus for osteogenic differentiation at this point in time (Figure 7).

Together, the biochemical quantification of calcium and phosphorus release and ALP enzymatic activity, combined with the transcriptional profile of *Runx2*, *Osterix*, ALP and *OPN*, provide a multi-level (transcriptional and protein/biochemical) chain of evidence for osteogenic modulation by the collagen membranes. Spatially resolved histochemical assays—ALP cytochemical staining and Alizarin Red S/Von Kossa mineralization staining on hFOB 1.19 cells—were not performed in the present screening study and are proposed as a complementary approach in future work to directly visualize mineralized nodule formation at the single-cell level.

#### 2.2.3. Osteogenic Gene Expression

The experimental results were obtained in hFOB 1.19 cells (human fetal osteoblasts) cultured in vitro on different collagen materials. The results demonstrate differential modulation of gene expression of key osteoblast differentiation markers (*Runx2*, *Osterix*, ALP, and *OPN*) in cells exposed to the different biomaterials (Biocollagen^®^, Derma^®^, and VantyColl^®^) compared to the untreated control (normalized to 1.0-fold).

For all genes analyzed (*Runx2*, *Osterix*, ALP and *OPN*), total RNA was extracted from hFOB 1.19 cells after 14 days of culture on each collagen membrane, a single time point selected based on the proliferation and biochemical mineralization kinetics observed in the preceding assays (Figure 5, Figure 6 and Figure 7).

All materials induced statistically significant changes (* *p* < 0.05, ** *p* < 0.01 vs. control), with gene-specific profiles reflecting sequential stages of osteogenesis.

##### *Runx2* Gene Expression

**Figure 8:**
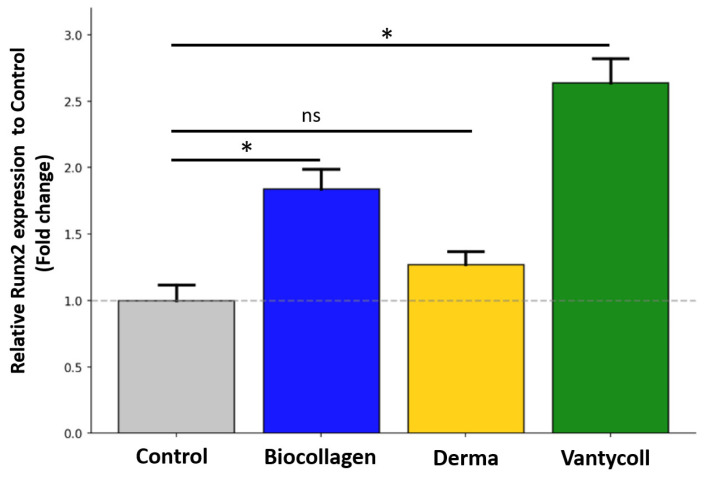
*Runx2* showed upregulation in all biomaterials. VantyColl^®^ showed the greatest effect, indicating early activation of the osteogenic lineage. Materials facilitate the initial mesenchymal–osteoblastic transition through adhesion signals and surface topography. * *p* < 0.05, ns, not significant. An asterisk (*) indicates that there are statistically significant differences (*p* < 0.05) compared to those without an asterisk.

*Runx2* (an early master transcription factor of osteoblastic cells) showed upregulation in all biomaterials. Biocollagen^®^ induced a 1.84-fold increase, Derma^®^ a 1.27-fold increase, and VantyColl^®^ the greatest effect at 2.64-fold**, indicating early activation of the osteogenic lineage, particularly by VantyColl^®^. These values suggest that collagenized materials facilitate the initial mesenchymal–osteoblastic transition through adhesion signals and surface topography (Figure 8).

##### *Osterix* Gene Expression

**Figure 9:**
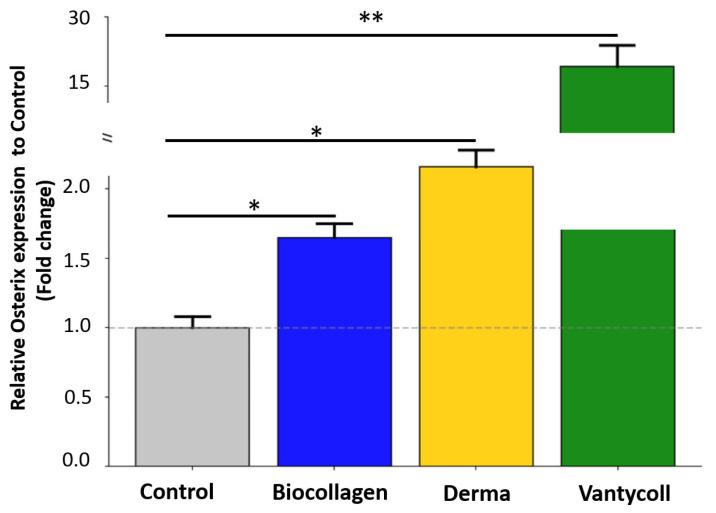
*Osterix* exhibited heterogeneous patterns. Biocollagen^®^ and Derma^®^ showed upregulation but VantyColl^®^ caused extreme upregulation: the highest value observed. This disparity highlights the selective osteoinductive potential of VantyColl^®^. * *p* < 0.05, ** *p* < 0.01.

*Osterix* (Sp7) (a downstream regulator of *Runx2* that is essential for proliferation and intermediate osteoblast maturation) exhibited heterogeneous patterns. Biocollagen^®^ showed a 1.65-fold* upregulation and Derma^®^ a 2.16-fold* upregulation, but VantyColl^®^ caused an extreme upregulation of 24.93-fold**, the highest value observed. This disparity highlights the selective osteoinductive potential of VantyColl^®^, possibly mediated by the release of bioactive factors or a porous microstructure that amplifies Smad/*Runx2*-*Osterix* pathways (Figure 9).

##### *OPN* Gene Expression

**Figure 10:**
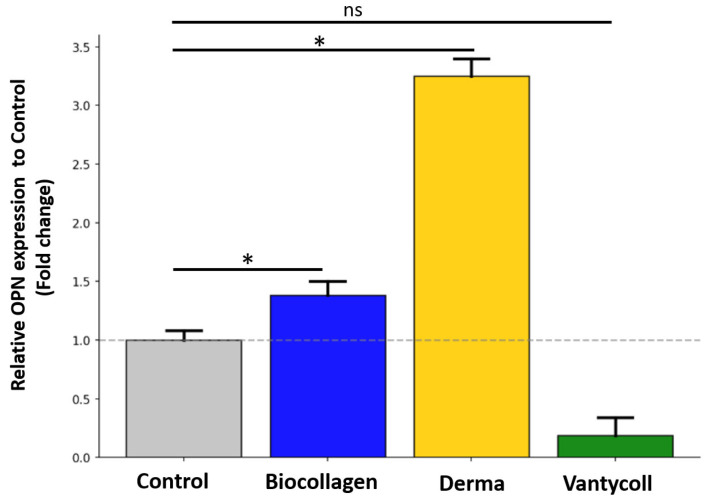
Osteopontin showed moderate upregulation in Biocollagen^®^ and Derma^®^, but strong suppression in VantyColl^®^. Derma^®^ stands out as the most effective for this final phase, suggesting its suitability for complex matrix environments. * *p* < 0.05, ns, not significant.

Osteopontin (*OPN*/SPP1) (an extracellular matrix glycoprotein associated with late maturation, inhibition of apoptotic mineralization, and bone remodeling) showed moderate upregulation in Biocollagen^®^ (1.38-fold*) and Derma^®^ (3.25-fold**), but strong suppression in VantyColl^®^ (0.19-fold). Derma^®^ stands out as the most effective for this final phase, suggesting its suitability for complex matrix environments (Figure 10).

##### ALP Gene Expression

**Figure 11:**
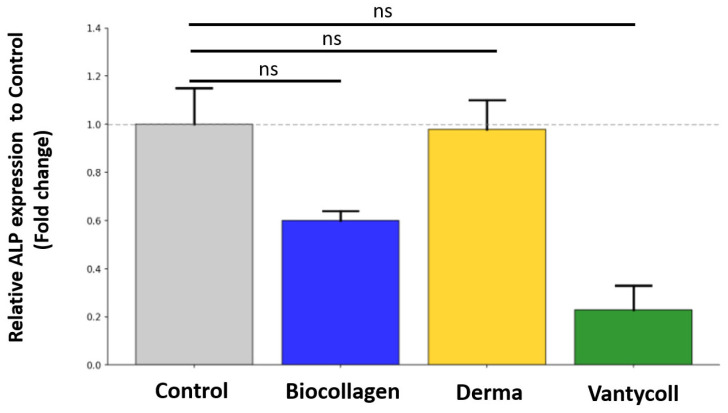
Alkaline phosphatase was consistently suppressed in all treated groups. VantyColl^®^ was the most pronounced reduction. This overall downregulation indicates that the biomaterials prioritize early proliferative phases over terminal mineralizing differentiation. ns, not significant.

Alkaline phosphatase (ALP), an enzymatic marker of early mineralization and matrix deposition, was consistently suppressed in all treated groups. Biocollagen^®^ showed a 0.60-fold reduction, Derma^®^ a 0.97-fold reduction, and VantyColl^®^ the most pronounced reduction at 0.23-fold. This overall downregulation indicates that the biomaterials prioritize early proliferative phases over terminal mineralizing differentiation, a common phenomenon in collagenized scaffolds that modulate osteoblastic phenotypic in vitro (Figure 11).

## 3. Discussion

Bone tissue engineering requires biomaterials that are not only biocompatible but also provide the optimal structural scaffolding and biochemical signals to guide cell proliferation and differentiation [14,15]. In GBR, the membranes must possess adequate rigidity, with resistance to collapse, in the application area to maintain sufficient space. This allows for the creation and preservation of a stable space necessary for optimal bone regeneration. From a clinical point of view, the GBR membrane must be very manageable by the dentist and have mechanical properties in its design to achieve a safe and effective fixation, especially to adapt it to irregular bone defects [16,17].

The main objective of this study was to evaluate three collagen membranes (Biocollagen^®^, Derma^®^, and VantyColl^®^) for their ability to support the culture of osteoblastic cells and human amniotic epithelial cells (hAECs), which have osteogenic potential. We aimed to study the influence of collagen microarchitecture on cell interaction. The scanning electron microscopy (SEM) results revealed significant differences in the microarchitecture of the membranes, which is fundamental to cell fate.

In fact, the Biocollagen^®^ membrane exhibited a lamellar morphology with a dense structure and a relatively smooth surface. This morphology, while ensuring structural integrity, may limit three-dimensional cell penetration and initial anchoring. The VantyColl^®^ membrane, for its part, exhibited high porosity and an interconnected fibrous network. High porosity and interconnected pore size have been shown to initially favor nutrient diffusion and rapid cell infiltration, promoting high early metabolic rates. Finally, the Derma^®^ membrane showed a clearly fibrillar and oriented architecture.

It is important to note that during GBR at the bone defect site, the collagen membrane surface is in direct contact with the grafted tissue. The membrane’s physical and chemical properties can influence cell–matrix and tissue interactions, which can affect essential processes such as cell migration, proliferation, and the deposition of extracellular matrix (ECM) and mineralized matrix proteins. In this sense, membrane surface roughness plays a fundamental role in regulating cell behavior, including adhesion, migration, proliferation, and differentiation. Thus, rough surfaces positively regulate the expression of osteogenic factors that significantly enhance osteoblast adhesion and osteogenesis [18,19].

A recent study analyzed, using optical microscopy and SEM, the macro- and microscopic characteristics of five different absorbable membranes used for GBR [18]. Under optical microscopy, all the membranes appeared rough, with ridges and depressions. Under SEM, most of the membranes showed a three-dimensional collagen fiber architecture. Pore size, density, and surface roughness, as well as the type of cross-linking, can influence cell interaction and lead to different regenerative processes, potentially improving the quality and time of tissue regeneration [20].

It is well documented that fiber orientation can serve as a guide for cell migration (a phenomenon known as contact guidance) and elongation. This guidance is positively correlated with the maintenance of long-term viability. Furthermore, fibrillar alignment has been shown to directly influence cell differentiation by modulating cell contractility and mechanosensitivity [21,22]. The aligned structure of the fibers not only improves cell adhesion and migration but also acts synergistically with the physicochemical properties of the membrane material. Furthermore, they found that this alignment enhances cell migration. The aligned fibers generate greater mechanical stress, and through mechanotransduction, this stress increases integrin expression and activates the ROX-2 signaling pathway, thus promoting osteogenic differentiation. In general, rapid cell migration to damaged areas is vital for effective tissue regeneration [22,23].

Subsequently, we evaluated cell proliferation and the temporal dynamics of cell lines on the different membranes using the MTT proliferation kit. Proliferation analysis demonstrated that all membranes were highly biocompatible, outperforming the control in proliferation for both cell types at most time points. In osteoblastic cells, the Derma^®^ membrane proved to be the most efficient in the long term, maintaining the highest proliferation rates on day 7 (approximately 2.5 times that of the control). This superior performance is likely linked to the stability and optimal support provided by its dense, oriented, fibrillar structure. In hAEC culture, a distinct pattern was observed: proliferation on the VantyColl^®^ membrane (initially very high) decreased sharply by day 7, falling to levels that were not significant compared to the control. This late collapse, despite its favorable porous morphology for initial adhesion, suggests that the material’s structure or composition may lack the necessary signals for long-term hAEC viability in a 3D environment, or that the high initial proliferation rate led to rapid nutrient depletion or contact inhibition.

Cell adhesion is the first critical step in tissue regeneration, as it directly determines subsequent cell extension, proliferation, and differentiation. Membrane structure can induce spatially differential adhesion and infiltration behaviors. In membranes with a porous and rough surface, cells can spread freely within the three-dimensional pore network, exhibiting complex stellate or polygonal morphologies, and their spread area increases significantly. This morphological difference may be due to the rough topography of the membrane surface, which provides abundant sites for multipoint attachment of cell pseudopods and cell body expansion. This rough and porous surface is conducive to cell adhesion and proliferation that guides osteogenesis for the GBR [24,25]. It should be noted that, in the present study, the influence of membrane microarchitecture on cell adhesion and spreading was inferred from proliferation kinetics and biochemical/molecular functional read-outs rather than from direct visualization of cell morphology on the membrane surface. Complementary techniques such as live/dead assays and cytoskeletal (F-actin/DAPI) fluorescence staining would provide direct visual confirmation of the spreading and adhesion patterns proposed here and are planned as part of a dedicated follow-up study. In fact, collagen membranes provide three-dimensional support that mimics the natural extracellular matrix, offering a favorable microenvironment for cell adhesion, proliferation, and differentiation. Hence, they have emerged as one of the primary supporting biomaterials utilized in tissue engineering for reconstructive therapies because of their exceptional biocompatibility and biodegradability [26,27].

In GBR, collagen barrier membranes create a microenvironment conducive to bone regeneration, blocking non-osteoblastic invasion while promoting osteoblast migration and differentiation. The autologous cellular component exhibits osteogenic activity, osteoinductivity, and osteoconductivity. Growth factors such as bone morphogenetic proteins and transforming growth factor β (TGF-β) regulate bone regeneration through cell signaling pathways. Technical advances include the harvesting of autologous bone, the use of absorbable collagen membranes, and bone substitutes that can be combined with autologous bone [27,28].

Finally, to study the osteoinductive capacity of each collagen membrane, we analyzed the concentration of Ca, P, and the enzymatic activity of alkaline phosphatase (ALP) in conditioned medium. This biochemical analysis of the conditioned medium is fundamental to demonstrating the membranes’ ability to guide cell differentiation. An increase in ALP activity is a key indicator of early osteogenic differentiation [28,29].

In osteoblastic cells, the VantyColl^®^ group induced peaks in ALP, calcium, and phosphorus activity on day 7. The strong correlation between these three markers suggests that the VantyColl^®^ membrane acts as a potent inducer of early osteogenic differentiation, possibly facilitated by its high porosity, which allows for rapid cell–material interaction and metabolite release. In contrast, the Biocollagen^®^ membrane in osteoblasts showed consistently low levels of calcium and phosphorus in the medium. A decrease in the concentration of inorganic ions in the medium during osteogenic culture can be interpreted as an indirect signal of ion consumption for mineral precipitation. This suggests that, while the lamellar structure of Biocollagen^®^ did not stimulate proliferation or ALP release to the same extent as other groups, it may be facilitating nucleation and the formation of a stable mineral phase in situ.

In hAECs, the ionic dynamics were more variable, reflecting their pluripotent nature. However, the calcium peak observed in Derma^®^ and the phosphorus peak in Biocollagen^®^ on day 7 suggest that these materials do actively interact with the amniotic cell to modulate mineral metabolism. In fact, in vivo implantation of loaded hAEC cells onto graft scaffolds not only enhanced bone regeneration through direct involvement but also reduced the early host immune response to the scaffolds. The hAEC cells possess adequate osteogenic differentiation potential and a modulatory influence on the early tissue remodeling process, making them a potential source of progenitor cells for the bone regeneration of alveolar defects [30]. The hAECs can significantly promote the proliferation, migration, and osteogenic differentiation of various cell lineages. Factors such as transforming growth factor β1 (TGFβ1) and microRNA 34a-5p (miR-34a-5p) are present in hAECs. Some research demonstrates that hAECs promote osteoblast differentiation through the secretion of TGFβ1 and miR-34a-5p, and that they could be an optimal cell source for bone regenerative medicine [31].

The osteogenic mechanisms of collagen membranes are not yet fully defined. The cellular responses and molecular mechanisms associated with tissue healing of different membranes have important implications for regulating bone regeneration through the properties of different materials. Furthermore, it is unclear whether different membranes exhibit similar cellular responses and molecular mechanisms in different hosts [28].

The collagen membranes evaluated differentially modulate the genetic cascade of osteogenesis (*Runx2* → *Osterix* → ALP → *OPN*), aligning with the temporal sequence established in in vitro models of osteoblastic differentiation [30]. The consistent upregulation of *Runx2* (1.27–2.64-fold), maximal in VantyColl^®^, confirms its pivotal role in initial mesenchymal involvement, and could plausibly be mediated by integrin–collagen interactions that activate FAK/PI3K pathways, although this mechanism was not directly investigated in the present study and remains hypothetical. Surprisingly, *Osterix* showed disproportionate induction in VantyColl^®^ (24.93-fold), exceeding thresholds reported in conventional collagenized scaffolds (2–5-fold). This outlier could hypothetically be attributed to the type I/III composition of VantyColl^®^, which may favor BMPR receptor clustering and Sp7-dependent transcriptional amplification, by analogy with mechanisms documented in resorbable membranes for guided bone regeneration (GBR) [32]; direct experimental confirmation of BMPR/Smad pathway involvement was not performed in this study and is proposed for future mechanistic investigation.

The universal suppression of ALP expression (<1-fold) with all biomaterials differs from studies with hydrolyzed collagen or chitosan–collagen, where 2- to 3-fold increases via MAPK/Erk1/2 are reported. This profile (*Runx2*/*Osterix* ↑, ALP ↓) suggests a transient “proliferative–osteogenic” state, optimized for prolonged cell viability in critical defects, rather than rapid mineralization. In parallel, the increased *OPN* in Derma^®^ (3.25-fold) supports its efficacy in the mature extracellular matrix, consistent with collagenized porcine xenografts such as Bio-Oss^®^/Osteobiol Gen-Os^®^, which exhibit superior osteoconductivity through sustained BMP-2/7 release [33].

It should be noted that ALP enzymatic activity (Figure 6 and Figure 7, measured in conditioned media at 1, 4, 7 and 14 days) and ALP gene expression (measured by qPCR at a single, later time point) reflect different biological levels and time frames, and their apparent divergence should not be interpreted as contradictory. ALP protein activity represents the cumulative product of earlier transcriptional activity and is subject to post-transcriptional and post-translational regulation (mRNA/protein stability, enzyme turnover), whereas ALP transcript levels at the later sampling time point analyzed here may already reflect the physiological down-regulation of ALP transcription that typically accompanies the transition from the proliferative/early-differentiation phase toward matrix maturation, as cells shift toward expression of late markers such as *OPN*. Methodological factors—a single time point for qPCR versus multiple time points for enzymatic activity—may also contribute to this apparent divergence. We therefore interpret the ALP data as reflecting a transient, time-dependent regulatory pattern rather than a genuine contradiction.

These functional differences underscore complementary clinical applications: VantyColl^®^ for GBR with an emphasis on rapid maturation (*Osterix*-dominant); Derma^®^ for volumetric grafts with matrix support (*OPN*-dominant); and Biocollagen^®^ as a balanced generic scaffold. These data strengthen the osteoinductive profile of commercial collagen membranes, with direct implications for regenerative dentistry and bone tissue engineering [34].

## 4. Materials and Methods

### 4.1. Sample Preparation

Fifteen collagen membranes with different structures (Biocollagen^®^; Derma^®^, VantyColl^®^) were provided by Bioteck S.R.L. (Arcugnano, Italy); Osteobiol^®^ (Tecnos, Giaveno, Italy) and Techbiomat^®^ (Barcelona, Spain), respectively (Figure 1, Figure 2 and Figure 3).

The composition and manufacturer-declared characteristics of the three collagen membranes evaluated are summarized in Table 1.

Prior to the use of the membranes for the experimental study, they were sterilized with ultraviolet rays and subsequently immersed in a solution of DMEN F12^®^ (Gibco Grand Island, New York, NY, USA) (Dulbecco’s modified Eagle medium/F-12 nutrient mixture) supplemented with 10% fetal bovine serum and 1% penicillin/streptomycin.

### 4.2. Characterization of Collagen Membrane Surfaces

To determine the structural characteristics of the membranes that could influence the cellular response, a detailed morphological analysis was performed using a Zeiss Crossbeam 550^®^ Scanning electron microscope (SEM) (Zeiss Group, Heidenheim an der Brenz, Germany) at different magnifications (350×, 3.00 K×, 12.00 K× and 30.00 K×).

### 4.3. Isolation and Culture of Human Amniotic Epithelial Cells (hAECs) and hFOB 1.19 Osteoblasts

Human placentas at term were obtained following written informed consent from healthy mothers after normal cesarean deliveries. All procedures used in this study were considered by the ethical committee of the hospitales universitarios Virgen Macarena-Virgen del Rocío de Sevilla (reference number SICEIA-2024-000860) and the patients signed consent forms for the research. All tested methods were conducted in accordance with relevant guidelines and regulations.

The amnion was mechanically separated from the chorion and washed with sterile physiological saline to remove blood and residual connective tissue. The tissue was cut into approximately 6 fragments of 8 cm × 8 cm and sterilized in a laminar flow through successive washing in sterile phosphate-buffered saline (PBS) containing penicillin/streptomycin 50 U/mL, amphotericin 50 U/mL, and cephalexin 50 U/mL. Subsequently, the amnion was digested with 0.25% trypsin-EDTA at 37 °C for 20 min. Amniotic epithelial cells (hAECs) were specifically released by this digestion. The remaining cells were centrifuged for 10 min at 1300 rpm. The cell pellet was resuspended and filtered through a sterile 100 μm filter (BD Falcon, Cox’s Bazar, Bangladesh) to eliminate tissue aggregates. Then, the cell solution was centrifuged for 10 min at 1300 rpm.

hFOB 1.19 human fetal osteoblasts (ATCC^®^ CRL-11372) were obtained from a certified cell bank and expanded under standard culture conditions (DMEM/F-12, 10% FBS, 1% penicillin/streptomycin, 37 °C, 5% CO_2_) prior to seeding onto the collagen membranes. hAECs were cultured for 24 h in fresh IMDM containing 10% FBS prior to seeding. At the end of each isolation procedure, the total number of isolated cells was determined.

Isolated cells were cultured in IMDM medium containing 10% FBS 4 mM L-glutamine, 1 mM sodium pyruvate, 50 U/mL penicillin, 50 μg/mL streptomycin, and 1 mM non-essential amino acids (Invitrogen, Carlsbad, CA, USA) at 37 °C in a 5% CO_2_ atmosphere. The medium was changed twice per week.

### 4.4. MTT Viability Assay

hAECs and hFOB 1.19 osteoblasts were seeded in 24-well plates onto the different collagen membranes (Biocollagen^®^, Derma^®^, VantyColl^®^) and on tissue-culture plastic as a membrane-free control (2 × 10^5^ cells per well). After the different time incubation, the cells were washed with PBS, and 80 μL of MTT (3-(4,5-dimethylthiazol-2-yl)-2,5-diphenyltetrazolium bromide) (final concentration 1 mg/mL) in 400 μL of DMEM-F12 medium containing 10% FBS was added. The cells were incubated for 30 min at 37 °C. In metabolically active cells, the mitochondrial enzyme succinate dehydrogenase converts MTT into formazan, a blue compound. To quantify the blue color generated, the medium was removed, and the formazan was dissolved in 200 μL of 100% ethanol, followed by incubation at room temperature for 5 min. The absorbance at 570 nm was measured using a DR-200Bs Microplate Reader spectrophotometer (Diatek, Kalyani, India).

### 4.5. Quantification of Alkaline Phosphatase Activity

Alkaline phosphatase (ALP) activity in hAEC and hFOB 1.19 osteoblast supernatants was quantified with Sensolyte pNPP alkaline phosphatase colorimetric assay (Anaspec, Fremont, CA, USA). The ALP activity was measured at 405 nm in a conventional ELx800 microplate reader (Bio-Tek^®^ Instruments, Inc., Winooski, VT, USA).

### 4.6. Calcium Intake

hFOB 1.19 osteoblast and hAEC supernatants were collected (1 mL) and stored at −80 °C. Calcium ion (Ca^2+^) concentrations were determined using the Calcium Assay Kit^®^ (Abcam, ab102505, Cambridge, UK) in accordance with the manufacturer’s protocol. In brief, supernatants were centrifuged at top speed for 5 min at 4 °C to eliminate any insoluble material. The resulting supernatants were then transferred to clean tubes. To each sample, 90 μL of the chromogenic reagent was added. The chromogenic complex formed between calcium ions and 0-cresolphthalein was quantified by measuring absorbance at 575 nm using a microplate reader. The absorbance values obtained for each standard were plotted against their final calcium concentration to create a standard curve. The calcium concentrations in the samples were determined by extrapolating from the standard curve. Finally, the calcium intake was calculated by subtracting the calcium concentration of each sample from the total control calcium concentration.

### 4.7. Quantitative Real-Time PCR Assay (qRT-PCR)

To investigate osteogenic gene expression, total RNA was isolated from hFOB 1.19 osteoblasts cultured on each collagen membrane for 14 days, using TRISURE^®^ reagent following the manufacturer’s instructions (Bioline GmbH, Berlin, Germany). The concentration and purity of the isolated RNA were determined spectrophotometrically at wavelengths of 260 nm and 280 nm in a NanoDrop ND-1000 Spectrophotometer^®^ (NanoDrop Technologies, Wilmington, NC, USA).

For complementary DNA (cDNA) synthesis, 5 μg of total RNA was subjected to reverse transcription at 50 °C for 1 h, employing the Transcriptor First Strand cDNA Synthesis Kit^®^ (Roche, Basel, Switzerland). Subsequently, a qRT-PCR reaction was carried out using forward and reverse primers designed specifically for the gene of interest (Table 2).

Quantitative RT-PCR (qRT-PCR) analysis was conducted using a SYBR Premix Ex Taq kit^®^ v (Takara BIO Inc., Shiga, Japan) and the PCR reactions were executed on an Applied Biosystems 7500 RT-PCR System^®^ (Applied Biosystems, Foster City, CA, USA). Each standard reaction consisted of 1 μM of both forward and reverse primers, 9 µL of SYBR Green master mix 2×, 1 μL of cDNA and water, resulting in a final reaction volume of 15 μL. The thermal cycling process consists of 95 °C for 10 min, 95 °C for 30 s, and 60 °C for 10 min for 40 amplification cycles, and annealing/extension at 60 °C for 1 min. The Opticon Monitor 3 Program was employed to determine the threshold cycle (CT) for each well. Relative quantification was determined using the 2^−∆∆CT^ technique. In the case of treated samples, the 2^−∆∆CT^ value indicated the fold change in gene expression, normalized against housekeeping genes (CYCLOPHILIN and GAPDH).

RNA integrity and purity were confirmed by a 260/280 nm absorbance ratio of 2.0. Primer specificity was confirmed by single-peak melt curve analysis for each gene, and amplification efficiency, calculated from standard curves of serially diluted cDNA, ranged between 90 and 110% for all primer pairs, consistent with MIQE guidelines. Cyclophilin were selected as reference genes based on their stable expression across the experimental conditions tested, and the geometric mean of both reference genes was used for normalization.

### 4.8. Statistical Analysis

Data are presented as mean ± standard deviation (SD) from n = 4 independent biological experiments. Each biological replicate (n = 4) corresponds to a distinct hAEC isolation (from four different placental donors, or at least four separate culture batches) and a distinct hFOB 1.19 passage/expansion seeded on a different day; no technical replicates were performed within each biological replicate, consistent with standard practice for this class of in vitro functional assay. Normality of data distribution and homogeneity of variances were assessed using the Shapiro–Wilk and Levene tests, respectively. For comparisons among the three membrane groups and the control at each individual time point, one-way analysis of variance (ANOVA) followed by Tukey’s post hoc test was applied. For the time-course datasets (proliferation, calcium, phosphorus and ALP activity), two-way ANOVA (membrane × time) with Tukey’s post hoc correction was additionally performed to evaluate main effects and membrane × time interactions. Statistical analyses were performed using GraphPad Prism version 11.0.0. Differences were considered statistically significant at *p* < 0.05.

### 4.9. Limitations

Although the present findings provide consistent evidence regarding the differential influence of collagen membrane microarchitecture on hAEC and hFOB 1.19 osteoblast behavior, several limitations should be acknowledged. First, this study is entirely in vitro; membrane performance in guided bone regeneration also depends on degradation kinetics, space-maintaining capacity, mechanical resistance, handling properties, and host tissue/immune responses, none of which were assessed here, and no animal or clinical data are yet available to confirm translation of these findings—in vivo validation was beyond the scope of the present investigation and should be addressed in future studies. Second, structural characterization of the membranes by SEM was qualitative; additional studies evaluating quantitative pore size, fiber diameter, porosity and surface roughness could further strengthen the understanding of the structure–function relationships proposed here. Third, direct visual evidence of cell adhesion and spreading (live/dead assay, cytoskeletal fluorescence staining, cell-seeded SEM) and histological mineralization assays (Alizarin Red S, Von Kossa) were not performed; osteogenic differentiation was instead assessed through the combination of proliferation, biochemical (ALP activity, calcium/phosphorus) and transcriptional (*Runx2*, *Osterix*, ALP, *OPN*) assays, which nevertheless provides robust, convergent evidence supporting the differential biological activity of the evaluated biomaterials. Fourth, molecular pathways discussed as candidate mechanisms (e.g., BMPR/Smad signaling, FAK/PI3K activation) were not experimentally investigated here and remain hypothetical. We consider these limitations to represent clear, actionable directions for future research rather than deficiencies that undermine the present conclusions, which are supported by convergent evidence across four independent, complementary assay types.

## 5. Conclusions

The results of this study suggest an association between the microarchitecture of the collagen membrane and the cellular response observed under the in vitro conditions tested; a causal relationship was not directly established. The Derma^®^ membrane (dense fibrillar structure) showed the best overall proliferative performance and stability, maintaining high proliferation and differentiation activity in both cell types until the end of the experiment. The VantyColl^®^ membrane (high porosity) was associated with a stronger early osteoblastic transcriptional and biochemical profile but showed reduced long-term hAEC viability under the conditions tested. These in vitro findings support the relevance of collagen membrane microarchitecture for cellular behavior in bone regeneration and highlight the potential relevance of selecting materials with ordered fibrillar architectures for applications requiring long-term tissue support, pending confirmation in further mechanistic and in vivo studies (see Section 4.9).

This experimental study suggests the importance of stem cells in bone regeneration of potential defects treated with guided bone regeneration techniques. In this regard, future research into the development of biomaterials containing amniotic stem cells for in vivo application in animals, and subsequently in clinical trials, would be interesting.

## Figures and Tables

**Table 1:** Composition and manufacturer-declared characteristics of the collagen membranes evaluated. Data compiled from manufacturer product documentation and the distributor’s technical literature; please verify against the specific product datasheets/lot certificates used in this study.

Membrane	Manufacturer	Source Tissue (Origin)	Collagen Type	Cross-Linking	Thickness	Est. Resorption	Main Clinical Indications
Biocollagen^®^	BIOTECK S.r.l. (Arcugnano, Italy)	Equine Achilles tendon	Type I	Non-cross-linked (enzymatic deantigenization)	≈0.2 mm	≈4–6 weeks	Protection/containment of granular bone grafts, alveolar ridge preservation, small socket/periodontal defect coverage
Derma^®^	Tecnoss S.r.l./ OsteoBiol^®^ (Giaveno, Torino, Italy)	Porcine dermis	Type I + III (bilayer, native fibers with elastin)	Non-cross-linked (native, cold-process)	0.5–2.0 mm (presentation-dependent)	≈1–5 months (thickness-dependent)	GBR with prolonged graft protection, ridge/socket preservation, epithelial exclusion barrier
VantyColl^®^	TechBiomat (Barcelona, Spain)	Equine (atelocollagen)	Type I	Non-cross-linked, lyophilized	Thin sheet, one smooth/one micro-rough side [AUTHORS TO CONFIRM]	≈10–12 weeks	Peri-implant bone defect protection, sinus-lift window coverage, socket coverage, sinus membrane perforation closure

**Table 2:** Sequences of the different gens studied.

Gene	Forward Primer (5′ → 3′)	Reverse Primer (5′ → 3′)
*ALP*	ACTCCCACTTCATCTGGAACC	CCTGTTCAGCTCGTACTGCAT
*RUNX2*	GTCTCACTGCCTCTCACTTG	CACACATCTCCTCCCTTCTG
*OSTERIX*	TGAGGAGGAAGTTCACTATGG	TTCTTTGTGCCTGCTTTGC
*OPN*	CAGAATGCTGTGTCCTCTGAA	GTCAATGGAGTCCTGGCTGT

## Data Availability

Raw data, original SEM micrographs and qPCR data will be made available upon reasonable request to the corresponding author.
